# Seasonal variation in the prevalence of acute undernutrition among children under five years of age in east rural Ethiopia: a longitudinal study

**DOI:** 10.1186/1471-2458-13-864

**Published:** 2013-09-18

**Authors:** Gudina Egata, Yemane Berhane, Alemayehu Worku

**Affiliations:** 1 Department of Public Health, College of Health Sciences, Haramaya University, P.O. Box: 235, Harar, Ethiopia; 2 Addis Continental Institute of Public Health, Professor of Epidemiology and Public Health, Addis Ababa, Ethiopia; 3 Department of Epidemiology and Biostatistics, School of Public Health, Doctor of Statistics Addis Ababa University, Addis Ababa, Ethiopia

**Keywords:** Acute child under-nutrition, Ethiopia, Under five children, Season

## Abstract

**Background:**

Malnutrition is a deficiency state of both macro and micronutrients (under - nutrition) and their over consumption (over- nutrition) causing measurable adverse effects on human body structure and function, resulting in specific physical and clinical outcomes. Little has been known about the seasonal variation in the magnitude of acute child under-nutrition and its determinants in low and middle-income countries making difficult the choice of a better nutrition intervention. The objective of this study was to determine the prevalence of acute under-nutrition and its associated factors on children aged 6 to 36 months in east rural Ethiopia in wet and dry seasons.

**Methods:**

A longitudinal study was conducted on children aged 6 to 36 months and their mothers (mother–child pairs) from July/August 2010/2011 to January/ February 2011/2012 in east rural Ethiopia. Data were collected from 2,132 mother–child pairs using a pretested structured questionnaire and the UNICEF recommended anthropometric measuring instruments after standardization. The Odds Ratio with 95% confidence interval was estimated to identify the predictors of acute child under nutrition (wasting) using a conditional fixed- effects logistic regression.

**Results:**

The prevalence of acute child under-nutrition was 7.4%; 95% CI: (6.3%, 8.5%) in wet and 11. 2%; 95% CI: (9.8%, 12.5%) in dry seasons. Child wasting was more common among children of poor households who had no cooperative bank saving accounts [AOR (95% CI) = 8.2. (1.8, 37.6)], and access to health facilities [AOR (95% CI) = 2.2 (1.4, 3.6)].

**Conclusion:**

Acute child under-nutrition was relatively higher in the dry season. Although season was not significantly associated with child under - nutrition, poverty and poor access to health services were important predictors of wasting in the study setting. Thus, effective community–based nutrition interventions that require a multi - disciplinary approach should be scaled up to curb childhood under-nutrition.

## Background

Malnutrition is a deficiency state of both macro and micronutrients (under - nutrition) and their over consumption (over- nutrition), causing measurable adverse effects on human body structure and function resulting in specific physical and clinical outcomes. Under-nutrition encompasses acute (wasting) and chronic (stunting) forms of malnutrition, and underweight together with deficiencies of other essential micronutrients [1-3].

Problems responsible for child under-nutrition are numerous. Some are basic problems, like political instability, slow economic growth, and lack of education. Others are underlying causes such as food insecurity and lack of maternal and child care services. The third groups include the highly specific risk factors like frequent infections and inadequate dietary intake [4,5]. Acute under –nutrition is often associated with short -term factors like seasonal variation in food availability, acute food shortages, shifts in social or economic policies, and the occurrence of illnesses beyond the expected level while its chronic form is associated with long- term risk factors. Moreover, underweight indicates either acute or chronic under-nutrition or both, and may be affected by either of the factors and is often used in the general evaluation of the health status of a population [6].

Child under - nutrition remains one of the major public health problems and the leading contributor to child morbidity and mortality in the world [5,7,8]. In the developing world, it is estimated that child under-nutrition is responsible for nearly 3.5 million deaths (54% of the under five mortality) [9], 35% of the disease burden in this age group, and 11% of the total global disability adjusted life years lost (DALYs) in these settings [10]. The overall risk of child death from preventable childhood diseases was 3.0 (2.0 - 4.5) times higher for moderate wasting, and 9.4 (5.3 –16.8) times higher for severe wasting [3]. In Ethiopia, severe and moderate malnutrition also accounts for 51% of the deaths of under five children of which 40% is due to moderate malnutrition [6].

About 10% (55 million children) aged under five is wasted worldwide of which the highest estimate (16%) is reported from South-central Asia. Out of these, 3·5% or 19 million children in developing countries are severely wasted with the highest proportion living in south- central and middle Africa [3]. On the other hand, between 36 and 41 million of these children are moderately wasted [11] and about 80% of them live in low - income countries [10] where malnutrition affects approximately one out of every three under five children [12].

Accordingly, different non-longitudinal studies conducted in resource poor settings including Sub-Saharan Africa (SSA) have showed that the prevalence of acute child malnutrition ranged from 3.68% to 25.66% [2,13-24]. On the contrary, only very few studies in these settings have identified the seasonal prevalence of acute child malnutrition and associated factors. For instance, there was a report on seasonal variation in the prevalence of acute child malnutrition at the end of the dry season (May/June) and at the end of the rainy season (October) indicating that the index for wasting (weight for length) was most sensitive to seasonal changes particularly to the end of the dry season unlike other indices of malnutrition which were less responsive to the seasonal changes [11,25,26].

Similarly, in another study, 16.5% of sedentary children aged less than 5 years were found to be acutely malnourished in rainy season while 10.6% of them have the problem in the dry season. Seasons, child age, mothers’ nutritional status, ethnicity, and place of residence were also identified as important predictors of seasonal acute child malnutrition [27]. In Ethiopia, the overall prevalence of wasting among under five children currently is 10%, out of which 3% are severely wasted [28,29].

However, in Ethiopia, no study has ever determined the magnitude of acute child under-nutrition and associated factors in different seasons to implement appropriate time oriented nutrition intervention. Therefore, this study was conducted to determine the prevalence of acute under-nutrition and associated factors in wet and dry seasons among children aged 6 to 36 months in rural east Ethiopia.

## Methods

The study was conducted in Kersa Demographic Surveillance and Health Research Centre (KDS-HRC) of Haramaya University, east Ethiopia. KDS-HRC is one of the demographic surveillance sites (DSS) in Ethiopia, comprising 10,256 households and a total adult population of 48,192 and 7,198 under five population (nearly 5% of the adult population) at the time of the study [30]. The DSS had three climatic zones, low land, high land, and midland. Climatic zone was one of the criteria on which the DSS was established. That is, kebeles (the smallest administrative units in Ethiopia) from each of the three climatic zones were represented in the site. The DSS was stratified into twelve rural kebeles and two semi - urban areas. In the study sites, there are health extension workers or community health workers who provide basic primary health care services, and the health care coverage of the district was 80% in 2010. The leading causes of morbidity among the under five children in the Out Patient Department of the study district were pneumonia, non-bloody diarrhea, and acute upper respiratory tract infections. On the other hand, the prevalence of HIV was 0.8% among the adult population, with no data on the under five population [31,32].

In some studies conducted in Ethiopia seasons were classified into four: spring (September – November), winter (December – February), Autumn (March - May), and Summer (June – August) to identify the prevalence of infectious diseases. The first two classifications above are predominantly dry seasons while the second two are wet seasons [33]. In this study, seasons were reclassified into two major categories: the dry season (December to May) and the wet season (June to November) because they are believed to have an association with the nutritional status of children.

Agriculture is the main livelihood earner of the study population. Crop production is basically on annual basis, except in few locations where it is biannual. Sorghum and maize are the common grains cultivated in the district. Some potato and other vegetables are also produced. Subsistent crops are often planted during the wet season and harvested during the dry season. Khat, a stimulant plant with amphetamine like effects, is predominantly produced as a cash crop. Polygamous marital relationship is common, and there are no profound cultural taboos related to eating habits [34].

A longitudinal study was conducted on children of 6 to 36 months of age (mother – child pair) during the wet and the dry seasons. The sample size was initially computed for the first objective which was to determine the prevalence of acute child under- nutrition in different seasons using a single population proportion formula with the following assumptions: 95.0% level of confidence, prevalence of underweight in rural settings of Oromiya (one of the administrative regions in the country) to be 34.0%, margin of error (desired precision between sample and population parameter) of 2%, and a contingency of 10.0% for possibilities of non–response [35]. Accordingly, a total of 2,371 sample of mother-child pairs was obtained. Secondly, sample size was estimated to assess factors associated with child under- nutrition during different seasons, using two population proportions formula to achieve an adequate power with the following assumptions: 95.0% level of confidence, 90% power, proportion of acute child malnutrition at the end of dry season 17%, proportion of acute malnutrition at the end of wet season 11%, and 20% non-response rate yielding a total of 1,685 child–mother pair [27]. Finally, the largest sample size was considered in this study.

The study participants were recruited from the kebeles under the DSS. Initially, the households in each kebele were selected using simple random sampling from the sampling frame of the KDS- HRC proportional to their estimated under five population size. The study population was then drawn from the randomly selected households in each study kebele/village proportional to the maximum sample size allocated for the study. If more than one under five children lived in the selected household, one child was selected by the lottery method. Data were obtained from the same mother - child pair at the base-line and at the end of the follow-up.

A structured and pretested questionnaire was used to collect data on background information from the mothers and their children. The questionnaire was initially prepared in English and translated into the local language, ***“Afan Oromo”***, by fluent speakers of both languages to maintain its consistency. Data collectors and supervisors were obtained from KDS - HRC and the surrounding community. Both categories received intensive training for one week on the questionnaire and interviewing techniques. Data collectors were paired during data collection to ensure data quality. The data collection process was supervised by trained supervisors who were nurses by profession. The data collection tool was pre-tested in a neighboring kebele outside the study area.

Data were collected at the baseline during the peak level of the wet season from July to August2010/2011 and in January and February 2011/2012 in the dry season at the end line of the study. Anthropometric measurements were taken after proper training and standardizing procedures. A pair of data collectors was assigned to interview a mother – child pair and take anthropometric measurements. A digital scale designed and manufactured under the guidance of UNICEF with 100 gram precision was used to measure body weight. Length/height measurements were taken using a locally produced UNICEF measuring board with a precision of 0.1 cm. Children below 24 months of age were measured in a recumbent position, while standing height was measured for those who were 24 months and older. Anthropometric measurements were taken twice and variations between the two measurements of 100 gram were accepted as normal for weight and 0.1 cm for height/length of the children. However, repeated measurements were carried out upon significantly larger variations [36]. Children were also evaluated for the presence or absence of oedema of the feet.

The outcome variable, the nutritional status of the children 6–36 months of age, was defined as follows. Under-nourished children were those with Weight for Height Z- score (WHZ) of less than −2 SD (Standard Deviation) using the new WHO child growth standards, while those children with a WHZ score equal to or greater than −2 SD were considered normal.

Maternal age, maternal literacy status and access to health facility, paternal occupation, data collection season, ownership of a cooperative bank saving account at household level, food security status, methods of solid waste disposal management, method of storing drinking water, child’s sex, and the number of under five children in the household were the main independent variables for this study.

Maternal age was grouped into “15-34 years” and coded “1” and into “35-49 years” and coded “0” on the basis that the more a mother gets old and becomes multiparous, the more she will be experienced in caring for the child in all aspects to prevent malnutrition. Maternal education was grouped as “illiterate” and coded “1” if the mother can’t read and write and coded “0” otherwise. This classification was preferred to other various levels of literacy as the majority of the rural mothers were illiterate. Literacy status was self reported and then confirmed by the data collector by giving the mother a text written in a local language.

The household heads were asked whether they had a cooperative bank saving account and coded “1” if they do not have an account and “0” otherwise. In this study, lack of a bank saving account implied that the household belonged to the poorer segment of the study population and otherwise. Paternal occupation was grouped as “farmers” and coded “0” and as “others” and coded “1”. Other categories included civil servants, owners of mini-private business, and daily laborers.

The household food security status was determined based on nine standard Household Food Insecurity Access Scale (HFIAS) questions adapted for this purpose from Food and Nutrition Technical Assistance (FANTA), 2007. The respondents were asked about the amount and variety of meal eaten, and the occurrence of food shortage for the household members, causing them not to eat the whole day or eat at night only, in the past four weeks preceding the survey [37]. All “Yes” responses were coded as’ one and “No” responses were coded as zero, and the responses were summed to produce an index of household food insecurity. The index had a high internal consistency (Cronbach’s alpha = 0.90) [38]. Later on, food secure households were coded “0” and food insecure ones “1” for further analysis.

Maternal access to health facility was categorized as “yes” and coded “0” and as “no” and coded “1”. In this study, access to health care facility was based on a proxy report of mothers to their main source of health care services, and the report of key informants in the respective kebeles of the study setting. It was also indicated that according to the principle of primary health care, accessibility refers to the availability of health care facilities for the clients within 10 km radius [32].

Data were double entered onto EPI- Data Version 3.1 and were exported to STATA version 11 for further analysis. The proportions of the independent variables were calculated against the season of data collection to check whether there was a seasonal variation within study participants. Anthropometric data were used to calculate WHZ-score using the WHO Anthro2010 software to determine children’s nutritional status.

The continuous variables such as maternal and child age were tested for normal distribution using some statistical tests including Kolmogorov - Smirnov and Shapiro -Wilk tests and through visual assessment using the normal curve with a histogram. The explanatory variables were tested for multi – collinearity using the Variance Inflation Factor (VIF) and the tolerance test. The VIF result for all models ranged between 1.00 – 1.27 while the tolerance test. was less than one which was within the normal limits [39]. The explanatory variables were tested for the interaction being in pair using appropriate STATA command and none of them showed the presence of an interaction between them.

Hausman’s test was used to choose among conditional fixed-effects and random - effects logistic regression model for the panel data analysis. Accordingly, the test was found to be statistically significant, supporting the use of the conditional fixed - effects logistic regression model [40].

Summary statistics, mean and standard deviation, were computed for the continuous variables. Paired t-test for dependent samples was used to check the mean weight difference for children and WHZ-score during the two seasons. Variability within individual study participants with respect to explanatory variables was compared using the conditional fixed - effects logistic regression model.

Crude analysis was conducted to check the association between dependant variable and different explanatory variables. All variables with a p value of ≤ 0.2 were considered for the multivariable analysis. The variables that showed a significant association with the outcome variable at bivariate analyses were entered together into the multivariable conditional fixed - effects logistic regression model to control all possible confounders and to identify the predictors of acute child under-nutrition. However, child sex, the known determinant of child under - nutrition, was considered regardless of its p value. Odds ratios with 95% confidence intervals were estimated and p - values were determined.

The study was cleared by the Ethical Review Committee of Haramaya University, College of Health Sciences, Ethiopia. Informed verbal and written consent was obtained from the parents/care givers of the children before the interview. Illiterate mothers consented by their thumb print after verbal consent.

## Results

Out of the initial 2352 mother–child pairs of the study participants recruited, only 2,234 of them participated in both surveys. One hundred and two mother–child pairs who either had incomplete data or flagged anthropometric measurements were excluded from the analysis. Thus, further analysis was conducted on 2132 mother – child pairs, making a response rate of 95.4%. The prevalence of acute child under-nutrition was 7.4%; 95% CI: (6.3%, 8.5%) in the wet season and 11.2%; 95% CI: (9.8%, 12.5%) in the dry season. The seasonal mean weight difference was 1.1 kg (± 3.0 SD) (p=0.001) which indicates that there was a significant change in the weight of the children (Table 1).

**Table 1:** Time variant characteristics of study participants by data collection season, Kersa district, east rural Ethiopia, 2011

**Characteristics of study participants**		**Wet season**		**Dry season**	
		**Number**	**%**	**Number**	**%**
Mother’s age	15-34	1668	78.79	1668	78.79
	35-49	449	21.21	449	21.21
Possession of a bank saving account at household level	No	2059	96.6	2060	96.6
	Yes	73	3.4	72	3.4
Household food security status	Food secure	1182	55.4	1401	65.71
	Food insecure	950	44.6	731	34.29
Children’s nutritional status	Normal	1975	92.64	1894	88.84
	Malnourished	157	7.36	238	11.16
Maternal weight (Kg)	Mean (±SD)	50.1 (6.35)		50.8 (5.98)	
Children’s age	Mean (±SD)	23.8 (9.07)		29.34 (9.22)	
Children’s weight (Kg)	Mean (±SD)	10.29 (2.13)		11.35 (2.17)	
Children’s MUAC (cm)	Mean (±SD)	13.80 (1.18)		13.85 (3.19)	
Children’s WHZ- score	Mean (±SD)	0.052 (1.37)		-.009 (1.72)	

In the multivariable conditional fixed- effects logistic regression model acute child under-nutrition was significantly associated with the lack of cooperative bank saving accounts among households [AOR (95% CI) = 8.2. (1.8, 37.6)], and poor maternal access to health facilities [AOR (95% CI) = 2.2 (1.4, 3.6)] (Table 2).

**Table 2:** Predictors of seasonal variation in the prevalence of acute child under-nutrition among children 6 to 36 months of age, Kersa district, east rural Ethiopia, 2011

**Characteristics**	**Category**	**AOR (95% CI)**
Mother’s age	35-49	1
	15-34	0.9 (0.5, 1.3)
Mother’s level of education	Literate	1
	Illiterate	1.6 ( 0.9, 2.9)
Paternal occupation	Farmers	1
	Others^∞^	0.8 (0.4, 1.7)
Possession of a bank saving account at household level	Yes	1
	No	8.2 (1.8, 37.6)**
Season	Dry	1
	Wet	1.6 (0.9, 2.8)
Household food security status	Food secure	1
	Food insecure	1.1. (0.7, 1.6)
Maternal access to a health facility	Yes	1
	No	2.2 (1.4, 3.6)**
Method of storing drinking water at household level	Properly stored	1
	Improperly stored	0.5 (0.2, 1.0)
Method of solid waste management	Managed properly	1
	Dumped	1.006 (0.6, 1.7)
Children’s sex	Female	1
	Male	1.2 (0.7, 2.0)
Number of under - five children in the household	Four per household	1
	> four per household	0.7 (0.5, 1.2)

** = p < 0.001 AOR = Adjusted odds ratio.

∞ = civil servant, daily laborer, owner of mini private business.

LRchi2 = 54.36, Prob > chi2 = 0.00001, Log likelihood = − 205.02335.

## Discussion

In this study, the prevalence of acute child under- nutrition was 7.4%; 95% CI: (6.25%, 8.5%) in wet and 11.2%; 95% CI: (9.8%, 12.5%) in dry seasons. Lack of a cooperative saving bank account and poor access to health facilities were also identified as strong predictors of acute child under – nutrition.

The rates of acute child under-nutrition observed during wet and dry seasons in this study are in agreement with what are seen in other non- longitudinal studies conducted in SSA countries [9,10,15-24] including Ethiopia [28,29]. This implies that acute child under- nutrition still continues to be a threat to the lives of under five children in resource constrained settings and that it requires due attention in order to successfully meet the MDGs related to child health in these countries.

In this research seasonal variations have been observed in the magnitude of acute child under – nutrition, with a relative rise of the condition in the dry season of the study setting. Regardless of this increase, the season was not significantly associated with acute child under- nutrition. The increase was found to be inconsistent with few documented longitudinal evidence from studies conducted in other developing settings other than Ethiopia [25-27]. In Ethiopia, no such evidence was documented from a panel study. This contrast in the finding could be attributed to the nature of the study setting where the cash crop, khat, was more popular than food crops [34]. It is also commonly known that the study district is food insecure regardless of the seasonal variation. The rate of food insecurity observed in this study could also support the former evidence related to food insecurity in the country [41]. Crop production is also not adequately diversified to satisfy the nutritional needs of the members of the households. As it has been observed during the field work, in most of the cases, mothers used to leave their small children at home with the older siblings who might not be competent to look after the young children even in the presence of food in the household. Thus, giving inadequate attention to child care by the mother (child ignorance) in the rural setup could also play a pivotal role in the prevalence of acute child under-nutrition.

Lack of cooperative bank saving account at a household level was significantly associated with acute child under-nutrition. There were few households who reported that they had bank saving accounts during the study period. The odds of acute child under-nutrition were seven times higher among children from the poorest households who did not have bank saving accounts during the study period. This could signal the deep rooted poverty in this study population. Other studies have also reported that childhood under - nutrition was more common among children in poor households [42-46].

Inadequate maternal access to health facilities was also significantly associated with child under- nutrition. Children whose mothers had less access to health facilities were twice more likely to be under- nourished compared with their counterparts. This is finding is similar to the findings of other studies in which the expansion of healthcare infrastructure significantly reduced the risk of child under-nutrition [46,47]. Nonetheless, maternal and child health service coverage was not satisfactory in this study setting [31].

Other known predictors of child under - nutrition, such as maternal education, age, paternal occupation, household food security status, and childhood morbidity in other similar studies turned out to be insignificant in this study [42,44]. This could be due to the homogeneity of the households with regard to some of their background constructs in this study. Mothers were also asked about the history of illness of their children for the past two weeks preceding each survey with the assumption that illness could aggravate the occurrence of childhood under- nutrition. Moreover, lack of significant association of children’s nutritional status with other known factors could be attributed to the analytical approach employed in this study, xtlogit (logistic regression for panel data), which is more powerful in controlling the correlation between the variables of the study [40].

The application of the conditional fixed-effects logistic regression model for panel data has also helped in controlling both characteristics of the study participants that change and do not change over time. In a panel analysis, the study participants serve as their own controls, and the effects of their characteristics are controlled for whether they are measured or not. The Fixed-effects models, unlike random-effects models, are thus less vulnerable to omitted variable bias even when one fails to measure certain variables. This is because the fixed- effects models estimate only within-individual differences discarding any information about differences between-individuals. It is the random- effects model that deals with the differences between-individual study subjects [40]. Moreover, this study was conducted in one of the DSS sites in the country where random selection of study participants for a study was possible. This could reduce the effect of selection bias and control the environmental factors, such as climatic variation which might have direct and indirect relationships with child under- nutrition. The standardization of the anthropometric measurements was also carried out to ensure the validity of the data collection procedures.

This study could have the following limitations. Firstly, as the information was gathered on the basis of the reports of mothers, it was likely to have a recall bias. In addition, the interview which dealt with household access to food for the last four months (using HFIAS questions) was also subject to an interviewer bias. Secondly, nutritional surveys were also liable to anthropometric measurement bias which could result in misclassification of children’s nutritional status. Nonetheless, the researchers conducted a standardization of the anthropometric instruments, provided an intensive training to the research team, and carried out a close supervision in the field to overcome the anthropometric measurement errors.

## Conclusion

According to the findings of this study, acute child under – nutrition was relatively high in the dry season. Although season was not significantly associated with child under - nutrition, household poverty and poor access to health facilities were important predictors of wasting. This indicated that acute childhood under-nutrition in the study area was strongly affected by household factors. Thus, effective community - based nutrition interventions that require a multi-disciplinary approach should be scaled- up to curb childhood under-nutrition.

## Competing interests

The authors declare that they have no competing interests.

## Authors’ contributions

GE participated in the design of the study, performed the data collection, performed the statistical analysis and served as the lead author of the manuscript. YB participated in the design of the study and contributed to finalization of the manuscript. AW participated in the design of the study, statistical analysis and in finalizing the manuscript. All authors read and approved the final manuscript.

## Authors’ information

GE is lecturer in the Department Of Public Health at Haramaya University, Ethiopia. YB is a senior professor of epidemiology and public health and director of the Addis Continental Institute of Public Health, Ethiopia. He has been teaching several courses in public health including, epidemiology and research methodology in various universities. He also has supervised many masters and doctoral students. He has more than 100 publications in national and international journals. AW is associate professor at Addis Ababa University, Ethiopia. He has been teaching biostatics and research methods in various universities for many years. He has more than 40 publications in peer reviewed national and international journals.

## Pre-publication history

The pre-publication history for this paper can be accessed here:

http://www.biomedcentral.com/1471-2458/13/864/prepub
